# Three new species of *Phanerochaete* (Polyporales, Basidiomycota)

**DOI:** 10.3897/mycokeys.41.29070

**Published:** 2018-10-26

**Authors:** Sheng-Hua Wu, Che-Chih Chen, Chia-Ling Wei

**Affiliations:** 1 Department of Biology, National Museum of Natural Science, Taichung 40419, Taiwan National Chung Hsing University Taichung Taiwan; 2 Department of Plant Pathology, National Chung Hsing University, Taichung 40227, Taiwan National Museum of Natural Science Taichung Taiwan

**Keywords:** China, corticioid fungi, multi-marker phylogeny, Phanerochaetaceae, Taiwan

## Abstract

*Phanerochaetecanobrunnea*, *P.cystidiata* and *P.fusca* are presented as new species, supported by morphological studies and two sets of phylogenetic analyses. The 5.8S+nuc 28S+*rpb1* dataset shows the generic placement of the three species within the phlebioid clade of Polyporales. The ITS+nuc 28S dataset displays relationships for the new taxa within *Phanerochaete* s.s. *Phanerochaetecanobrunnea* grew on angiosperm branches in subtropical Taiwan and is characterised by greyish brown hymenial surface, brown generative hyphae and skeletal hyphae and absence of cystidia. *Phanerochaetecystidiata* grew on angiosperm branches above 1000 m in montane Taiwan and SW Yunnan Province of China and is characterised by cream to yellowish hymenial surface and more or less encrusted leptocystidia. *Phanerochaetefusca* grew on angiosperm branches at 1700 m in Hubei Province of China and is characterised by dark brown hymenial surface, leptocystidia, brown subicular hyphae and colourless to brownish basidiospores.

## Introduction

The genus *Phanerochaete* P. Karst., typified by *P.alnea* (Fr.) P. Karst., belongs to the Polyporales of the Basidiomycota and encompasses, when taken in a broad sense (Eriksson et al. 1978; Burdsall 1985; Wu 1990), over 150 names (Index Fungorum 2018). *Phanerochaete* spp. are typically recognised by its membranaceous, effuse, smooth hymenial surface (some are tuberculate, odontioid-hydnoid or merulioid-poroid), mostly monomitic hyphal system, simple-septate generative hyphae or with rare clamp connections in the subiculum, clavate basidia and ellipsoid to cylindrical, thin-walled and smooth basidiospores, which are inamyloid and non-dextrinoid. *Phanerochaete* is widely distributed and occurs on twigs, branches or trunks of angiosperms or gymnosperms, causing white rot in wood.

*Phanerochaete* recently has been shown to be a polyphyletic group, containing members placed throughout the phlebioid clade of Polyporales (Binder et al. 2005; Wu et al. 2010; Floudas and Hibbett 2015; Miettinen et al. 2016; Justo et al. 2017). *Phanerochaete* s.l. comprises some segregate genera: *Efibula* Sheng H. Wu, *Hydnophlebia* Parmasto, *Phaeophlebiopsis* Floudas & Hibbett, *Phlebiopsis* Jülich, *Rhizochaete* Gresl., Nakasone & Rajchenb. and *Scopuloides* (Massee) Höhn. & Litsch. (Burdsall 1985; Wu 1990; Greslebin et al. 2004; Wu et al. 2010; Floudas and Hibbett 2015).

The field survey of the corticioid fungi from Taiwan and mainland China conducted in 2014, 2015 and 2017, have revealed three new species of *Phanerochaete* s.s. presented herein, based on morphological and phylogenetic evidence.

## Materials and methods

### Morphological studies

Voucher specimens are deposited at the herbarium of National Museum of Natural Science of ROC (TNM). We used three mounting media for microscopic studies: 5% potassium hydroxide (KOH) with 1% phloxine was used for observation and measurements; Melzer’s reagent (IKI) was utilised to determine amyloidity and dextrinoidity and Cotton blue (CB) was utilised to check cyanophily. A standard method of measurement for microscopic characters follows Wu (1990). Below abbreviations were used when presenting statistic measurements of basidiospores: L = mean basidiospore length, W = mean basidiospore width, Q = variation in L/W ratio, n = number of measured spores. The terminology of microscopic characters followed Wu (1990).

### DNA extraction and sequencing

Dried specimens or mycelia were first ground into a fine powder using liquid nitrogen and a TissueLyser II (Qiagen, Hilden, Germany). DNA was then extracted using the Plant Genomic DNA Extraction Miniprep System (Viogene-Biotek Corp., New Taipei, Taiwan) according to the manufacturer’s instructions. The rDNA ITS1-5.8S-ITS2 (ITS) was amplified using primer pairs ITS1/ITS4 (White et al. 1990). The D1-D2 domain of nuc 28S rDNA (nuc 28S) was amplified using primer pair LR0R/LR5 (http://www2.clarku.edu/faculty/dhibbett/Protocols_Folder/Primers/Primers.pdf). RNA polymerase II largest subunit (*rpb1*) was amplified using the primer pair RPB1-Af/RPB1-Cr (Stiller and Hall 1997; Matheny et al. 2002). Both RPB1-2.1f and RPB1-2.2f were used as alternative primers to pair with RPB1-Cr (Frøslev et al. 2005). The PCR protocols for ITS, nuc 28S and *rpb1* followed Wu et al. (2018). PCR products were directly purified and sequenced by the MB Mission Biotech Company (Taipei, Taiwan). We determined the identity and accuracy of newly obtained sequences by comparing them to sequences in GenBank and assembled them using BioEdit (Hall 1999). Newly obtained sequences were then submitted to GenBank (https://www.ncbi.nlm.nih.gov/genbank/; Table 1).

**Table 1.** Species and sequences used in the phylogenetic analyses. Newly generated sequences are shown in bold.

Taxon	Strain/Specimen	ITS (contains 5.8S)	nuc 28S	*rpb1*
* Bjerkandera adusta *	HHB-12826-Sp	KP134983	KP135198	KP134784
* Byssomerulius corium *	FP-102382	KP135007	KP135230	KP134802
* Candelabrochaete africana *	FP-102987-Sp	KP135294	KP135199	KP134872
* Ceraceomyces serpens *	HHB-15692-Sp	KP135031	KP135200	KP134785
* Ceriporia alachuana *	FP-103881-Sp	KP135341	KP135201	KP134845
* Ceriporia purpurea *	KKN-223-Sp	KP135044	KP135203	KP134788
* Efibula americana *	FP-102165	KP135016	AY684165	AY864873
* Emmia lacerata *	FP-55521-T	KP135024	KP135202	KP134805
* Gloeoporus pannocinctus *	L-15726-Sp	KP135060	KP135214	KP134867
* Hydnophlebia chrysorhiza *	FD-282	KP135338	KP135217	KP134848
* Hyphoderma litschaueri *	FP-101740-Sp	KP135295	KP135219	KP134868
* Hyphoderma mutatum *	HHB-15479-Sp	KP135296	KP135221	KP134870
* Hyphodermella rosae *	FP-150552	KP134978	KP135223	KP134823
* Meruliopsis alnbostramineus *	HHB-10729	KP135051	KP135229	KP134787
* Phaeophlebiopsis peniophoroides *	FP-150577	KP135417	KP135273	KP134813
* Phanerochaete aculeata *	Wu 880701-2	–	GQ470636	–
* Phanerochaete affinis *	KHL11839	EU118652	EU118652	–
* Phanerochaete alnea *	OM8110	KP135171	–	–
* Phanerochaete arizonica *	RLG-10248-Sp	KP135170	KP135239	KP134830
* Phanerochaete australis *	HHB-7105-Sp	KP135081	KP135240	KP134840
* Phanerochaete bambusicola *	Wu 0707-2	MF399404	MF399395	LC314324
* Phanerochaete brunnea *	He1873	KX212220	KX212224	–
* Phanerochaete burtii *	HHB-4618	KP135117	KP135241	KP134829
* Phanerochaete calotricha *	Vanhanen-382	KP135107	–	KP134826
*** Phanerochaete canobrunnea ***	**CHWC 1506-17**	**LC412093**	**LC412102**	–
	**CHWC 1506-39**	**LC412094**	**LC412103**	–
	**CHWC 1506-66**	**LC412095**	**LC412104**	–
* Phanerochaete carnosa *	HHB-9195-Sp	KP135129	KP135242	KP134831
* Phanerochaete chrysosporium *	HHB-6251-Sp	KP135094	KP135246	KP134842
* Phanerochaete citrinosanguinea *	FP-105385	KP135100	KP135234	KP134824
* Phanerochaete concrescens *	LE < RUS>:287,008	KP994375	–	–
* Phanerochaete cumulodentata *	H:6,033,465	LN833868	–	–
	VL212	JF440574	–	–
*** Phanerochaete cystidiata ***	**GC 1708-358**	**LC412096**	**LC412101**	**LC412107**
	**Wu 1708-326**	**LC412097**	**LC412100**	**LC412108**
* Phanerochaete ericina *	HHB-2288	KP135167	KP135247	KP134834
* Phanerochaete exilis *	HHB-6988	KP135001	KP135236	KP134799
*** Phanerochaete fusca ***	**Wu 1409-161**	**LC412098**	**LC412105**	**LC412109**
	**Wu 1409-163**	**LC412099**	**LC412106**	**LC412110**
* Phanerochaete incarnata *	WEI 16-078	MF399407	MF399398	LC314327
* Phanerochaete krikophora *	HHB-5796-Sp	KP135164	KP135268	KP134837
* Phanerochaete laevis *	HHB-15519-Sp	KP135149	KP135249	KP134836
* Phanerochaete livescens *	FD-106	KP135070	KP135253	KP134841
* Phanerochaete magnoliae *	HHB-9829-Sp	KP135089	KP135237	KP134838
* Phanerochaete odontoidea *	Wu 9310-8	MF399408	MF399399	LC314328
* Phanerochaete porostereoides *	He1902	KX212217	KX212221	–
	He1908	KX212218	KX212222	–
* Phanerochaete pseudomagnoliae *	PP-25	KP135091	KP135250	KP134839
* Phanerochaete pseudosanguinea *	FD-244	KP135098	KP135251	KP134827
* Phanerochaete rhodella *	FD-18	KP135187	KP135258	KP134832
* Phanerochaete robusta *	Wu 1109-69	MF399409	MF399400	LC314329
* Phanerochaete sacchari *	Wu 880313-6	–	GQ470654	–
* Phanerochaete sanguinea *	HHB-7524	KP135101	KP135244	KP134825
* Phanerochaete sanguineocarnosa *	FD-359	KP135122	KP135245	KP134828
* Phanerochaete sordida *	FD-241	KP135136	KP135252	KP134833
* Phanerochaete stereoides *	VPCI207312	KF291012	–	–
	Wu 9708-118	–	GQ470661	–
* Phanerochaete subceracea *	FP-105974-R	KP135162	KP135255	KP134835
* Phanerochaete subodontoidea *	Wu 0106-35	MF399411	MF399402	LC314331
* Phanerochaete taiwaniana *	Wu 0112-13	MF399412	MF399403	LC314332
* Phanerochaete thailandica *	2015_07	MF467737	–	–
* Phanerochaete velutina *	Kotiranta21402	KP135179	–	–
* Phlebia centrifuga *	HHB-9239-Sp	KP135380	KP135262	KP134844
* Phlebia chrysocreas *	HHB-6333-Sp	KP135358	KP135263	KP134861
* Phlebia fuscoatra *	HHB-10782-Sp	KP135365	KP135265	KP134857
* Phlebia radiata *	AFTOL-484	AY854087	AF287885	AY864881
* Phlebia uda *	FP-101544-Sp	KP135361	KP135232	KP134859
* Phlebiopsis gigantea *	FP-70857-Sp	KP135390	KP135272	KP134821
* Pirex concentricus *	OSC-41587	KP134984	KP135275	KP134843
* Rhizochaete radicata *	FD-123	KP135407	KP135279	KP134816
* Scopuloides rimosa *	HHB-7042	KP135350	KP135282	KP134853
* Terana caerulea *	FP-104073	KP134980	KP135276	KP134865

### Phylogenetic analyses

We included two datasets for phylogenetic analyses. The 5.8S+nuc 28S+*rpb1* was compiled for inferring generic classification of target species within the phlebioid clade of Polyporales. The ITS+nuc 28S was compiled for getting better resolutions on species level within *Phanerochaete* s.s. The selection of strains and species consulted Wu et al. (2010), Floudas and Hibbett (2015), Volobuev et al. (2015), Liu and He (2016), Miettinen et al. (2016) and Wu et al. (2018). MAFFT v. 7 was used to align sequences of each marker with default settings (Katoh and Standley 2013). The resulting alignments were manually adjusted in MEGA 7 (Kumar et al. 2016). *Hyphodermalitschaueri* (Burt) J. Erikss. & Å. Strid and *H.mutatum* (Peck) Donk, were chosen as the outgroup in the 3-marker dataset. *Phlebiopsisgigantea* (Fr.) Jülich was chosen as the outgroup in the 2-marker dataset. Final datasets were deposited at TreeBASE (submission ID 23083).

For both datasets, Maximum Likelihood (ML) and Bayesian Inference (BI) analyses were performed, respectively, using RAxML BlackBox (Stamatakis et al. 2014) and MrBayes v. 3.2.6 (Ronquist et al. 2012) at the CIPRES Science Gateway (Miller et al. 2010; http://www.phylo.org/). For BI analysis, jModeltest 2.1.10 (Darriba et al. 2012) was first carried out to determine the best-fit substitution model for each marker based on Akaike Information Criterion (AIC). The GTR+I+G was used as the substitution model for the entire alignment of the 3-marker dataset, while, for the 2-marker dataset, the HKY+I+G and the GTR+I+G were used for the alignments of ITS and nuc 28S, respectively. The parameters for BI analyses were as follows: ngen = 10000000, samplefreq = 100, nchains = 4, nst = 6 for GTR, nst = 2 for HKY, rates = invgamma, burn-in = 25000. Fifty percent majority-rule consensus phylograms with posterior probability values (PP) were obtained when the average standard deviation of split frequencies was below 0.001. For ML analysis, the best-scoring tree with values of bootstrap (BS) was constructed using the GTR model with one hundred rapid bootstrap inferences. Gaps were regarded as missing data. Phylograms were visualised and edited by TreeGraph 2 (Stöver and Müller 2010) and Adobe Illustrator (Adobe Systems, Inc).

## Results

### Phylogenetic analyses

**Figure 1.**
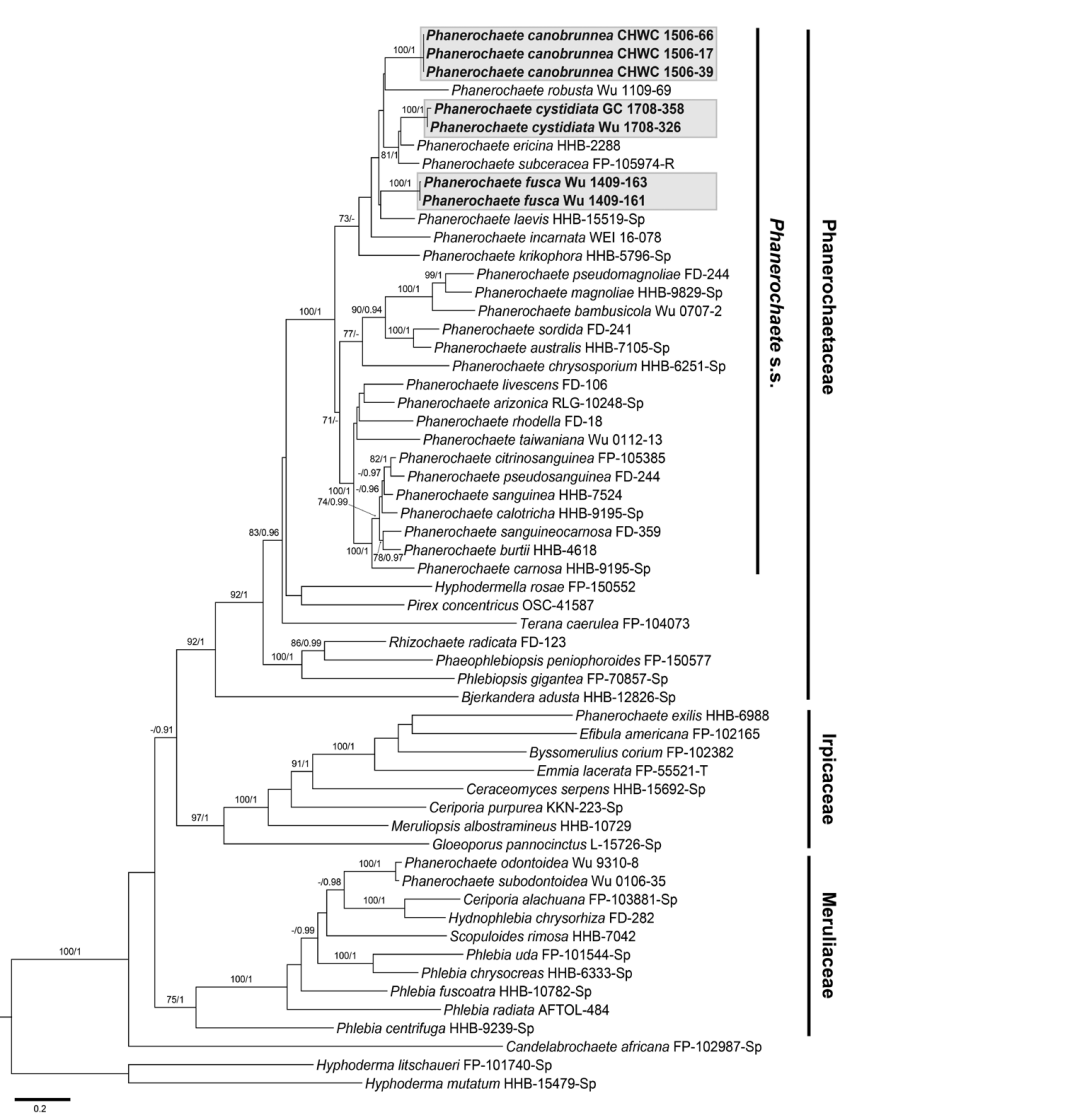
Phylogram inferred from Maximum likelihood analysis of the concatenated 5.8S+nuc 28S+*rpb1* dataset of representative taxa in the phlebioid clade of Polyporales. Branches are labelled with Maximum likelihood bootstrap values ≥70% and Bayesian posterior probabilities ≥0.9. Studied taxa are shaded with greyish boxes. Scale bar = substitutions per site.

**Figure 2.**
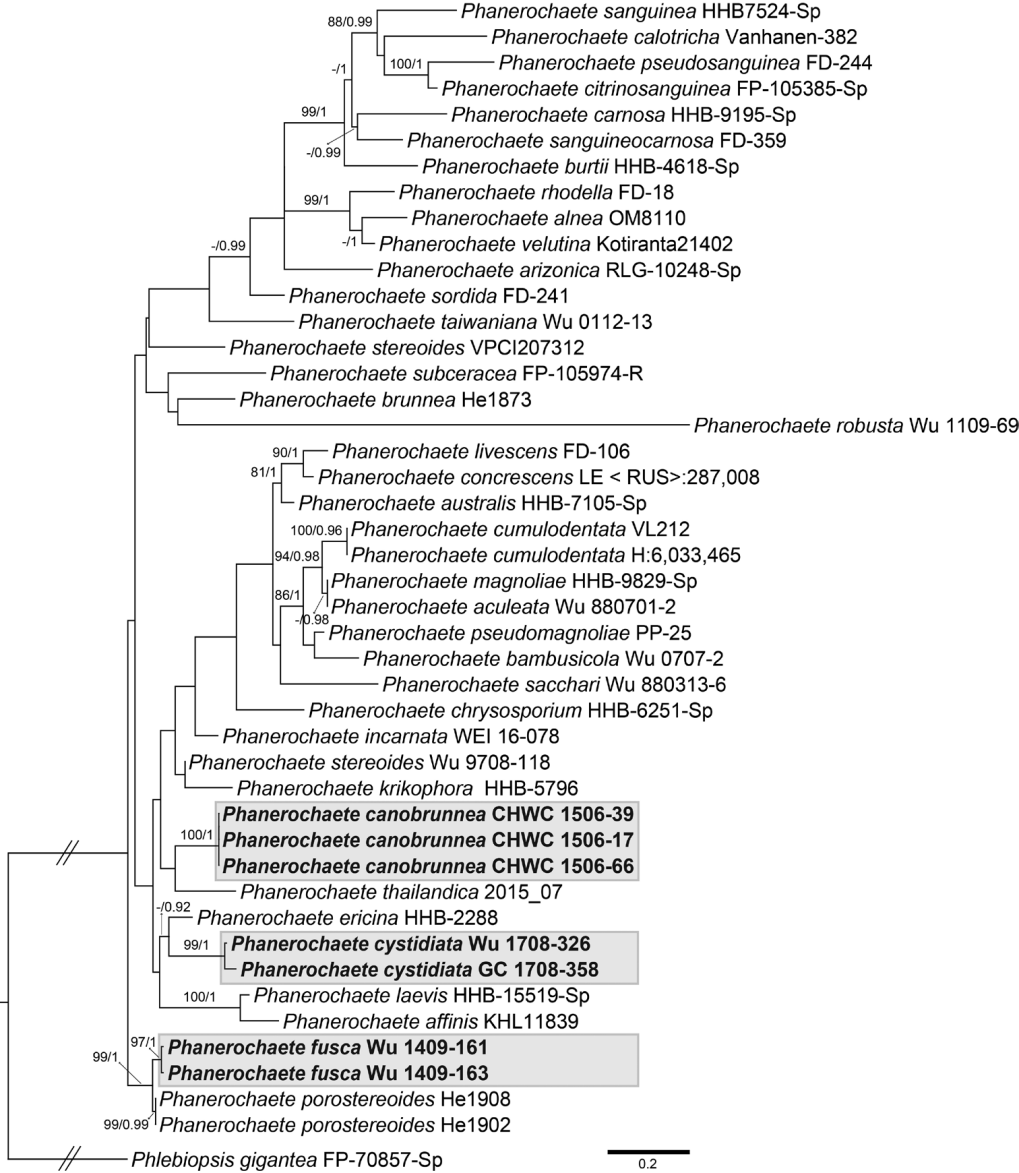
Phylogram inferred from Maximum likelihood analysis of the concatenated ITS+nuc 28S dataset of taxa in *Phanerochaete* s.s. Nodes are labelled with Maximum likelihood bootstrap values ≥70% and Bayesian Posterior probabilities ≥0.9. Studied taxa studied are shaded with greyish boxes. Scale bar = substitutions per site.

The 5.8S+nuc 28S+*rpb1* dataset consisted of 58 sequences of 2481 characters including gaps, of which 931 sites were parsimony informative. The ITS+nuc 28S dataset consisted of 45 sequences of 2199 characters including gaps, of which 220 sites were parsimony informative. Topologies of phylogenetic trees of each dataset inferred from BI and ML methods were similar and, thus, only ML trees were shown (Figs 1, 2). In the 3-marker analyses (Fig. 1), three main subclades of the phlebioid clade of Polyporales, annotated as three families, Irpicaceae, Meruliaceae and Phanerochaetaceae, could be recognised in the ingroup (BS = 75–97%, PP = 1). Sequences of three new species were nested within the lineage of *Phanerochaete* s.s. of Phanerochaetaceae (BS = 100%, PP = 1). In the 2-marker analyses (Fig. 2), sequences of each of three new species formed well-supported monophyletic group (BS = 97–100%, PP = 1). *Phanerochaetecanobrunnea, P.cystidiata* and *P.fusca* were allied to *P.thailandica* Kout & Sádlíková, *P.ericina* (Bourdot) J. Erikss. & Ryvarden and *P.porostereoides* S.L. Liu & S.H. He, respectively, based on available sequences.

### Taxonomy

#### Phanerochaete canobrunnea

Taxon classificationFungiPolyporalesPhanerochaetaceae

Sheng H. Wu, C.C. Chen & C.L. Wei sp. nov.

MycoBank No: 827411

Figs 3A , 4

##### Diagnosis.

*Phanerochaetecanobrunnea* is recognised by brown generative hyphae and brown skeletal hyphae, in combination with absence of cystidia.

##### Holotype.

TAIWAN. Nantou County: Yuchih Township, Lienhuachih, 23°55'N, 120°53'E, 715 m alt., on angiosperm branch, coll. W.C. Chen, C.C. Chen & C.L. Wei, 23 Jun 2015, *CHWC 1506*-*17* (TNM F0029207).

##### Etymology.

From canus+brunneus (= greyish-brown), referring to the colour of the hymenial surface.

##### Description.

Basidiome resupinate, effuse, loosely adnate, membranaceous, 250–500 μm thick in section. Hymenial surface pale greyish-brown, slightly darkening in KOH, smooth, sometimes cracked; margin concolorous or brownish, slightly fibrillose or determinate.

Hyphal system dimitic; generative hyphae mostly simple-septate, single or double clamp connections occasionally present in subiculum. Subiculum fairly uniform, with fairly loose texture, 200–400 μm thick; generative hyphae interwoven, brown, more or less straight, moderately ramified, rarely encrusted, 4–9 (–11) μm diam., thin- to thick-walled, walls up to 1.5 μm thick, anastomoses occasional; skeletal hyphae interwoven, brown, more or less straight, un-ramified or ramified, 2–5 μm diam., usually subsolid or thick-walled, walls up to 1.5 μm, adventitious septa occasionally present. Hymenial layer thickening, with dense texture, 50–100 μm thick; hyphae more or less vertical, brownish to subcolourless, 3–6 μm diam., thin-walled. Cystidia lacking. Basidia subclavate to clavate, 15–25 × 5–6 μm, 4-sterigmate. Basidiospores ellipsoid to narrowly ellipsoid, adaxially flattened, smooth, thin-walled, IKI –, CB –, mostly 4.2–5.8 × 2.5–3 μm. [(4–) 4.5–5.8 (–6) × (2.5–) 2.7–3 (–3.2) μm, L = 5.10±0.54 μm, W = 2.86±0.18 μm, Q = 1.78 (n = 30) (*CHWC 1506-17*); (4–) 4.2–5 (–5.8) × (2.3–) 2.5–2.8 (–3) μm, L = 4.63±0.42 μm, W = 2.66±0.17 μm, Q = 1.75 (n = 30) (*CHWC 1506-39*)].

##### Additional specimens examined (paratypes).

TAIWAN. Nantou County: Yuchih Township, Lienhuachih, 23°55'N, 120°53'E, 715 m alt., on angiosperm branch, coll. W.C. Chen, C.C. Chen & C.L. Wei, 23 Jun 2015, *CHWC 1506*-*39* (TNM F0029217); *CHWC 1506*-*66* (TNM F0029236).

##### Distribution.

Known from subtropical Taiwan.

**Remarks.** Amongst the few species in *Phanerochaete* having brown subicular hyphae, only *P.canobrunnea* and *P.thailandica* possess skeletal hyphae [described as “quasi-binding hyphae” in the protologue of *P.thailandica*, Sadlikova and Kout (2017)]. These two species are also closely related according to the phylogenetic analyses (Fig. 2). However, *P.thailandica* bears leptocystidia and has larger basidiospores (7–8 × 4–4.5 µm) (Sadlikova and Kout 2017). *Phanerochaetebrunnea* Sheng H. Wu resembles *P.canobrunnea* in lacking cystidia and having similar basidiospores, but its hyphal system is monomitic (Wu 1990). These two species are phylogenetically not closely related (Fig. 2).

**Figure 3.**
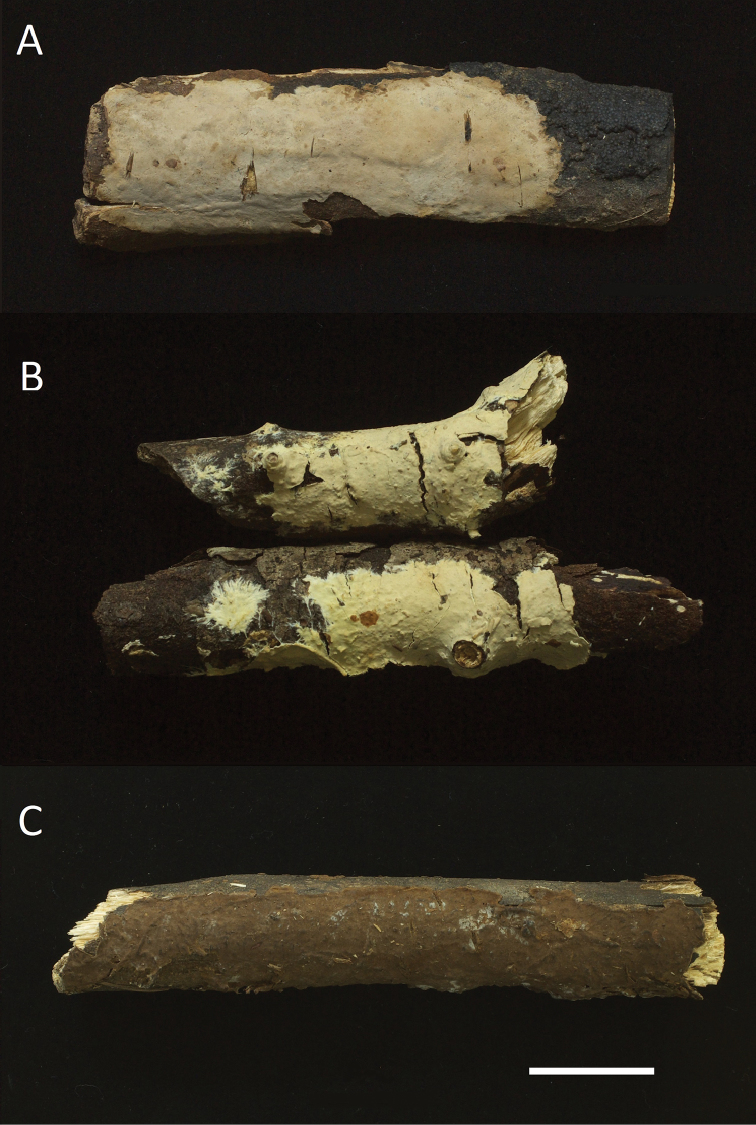
Basidiomes. **A***Phanerochaetecanobrunnea* (holotype, *CHWC 1506*-*17*) **B***P.cystidiata* (holotype, *GC 1708-358*) **C***P.fusca* (holotype, *Wu 1409*-*161*). Scale bar:1cm.

**Figure 4.**
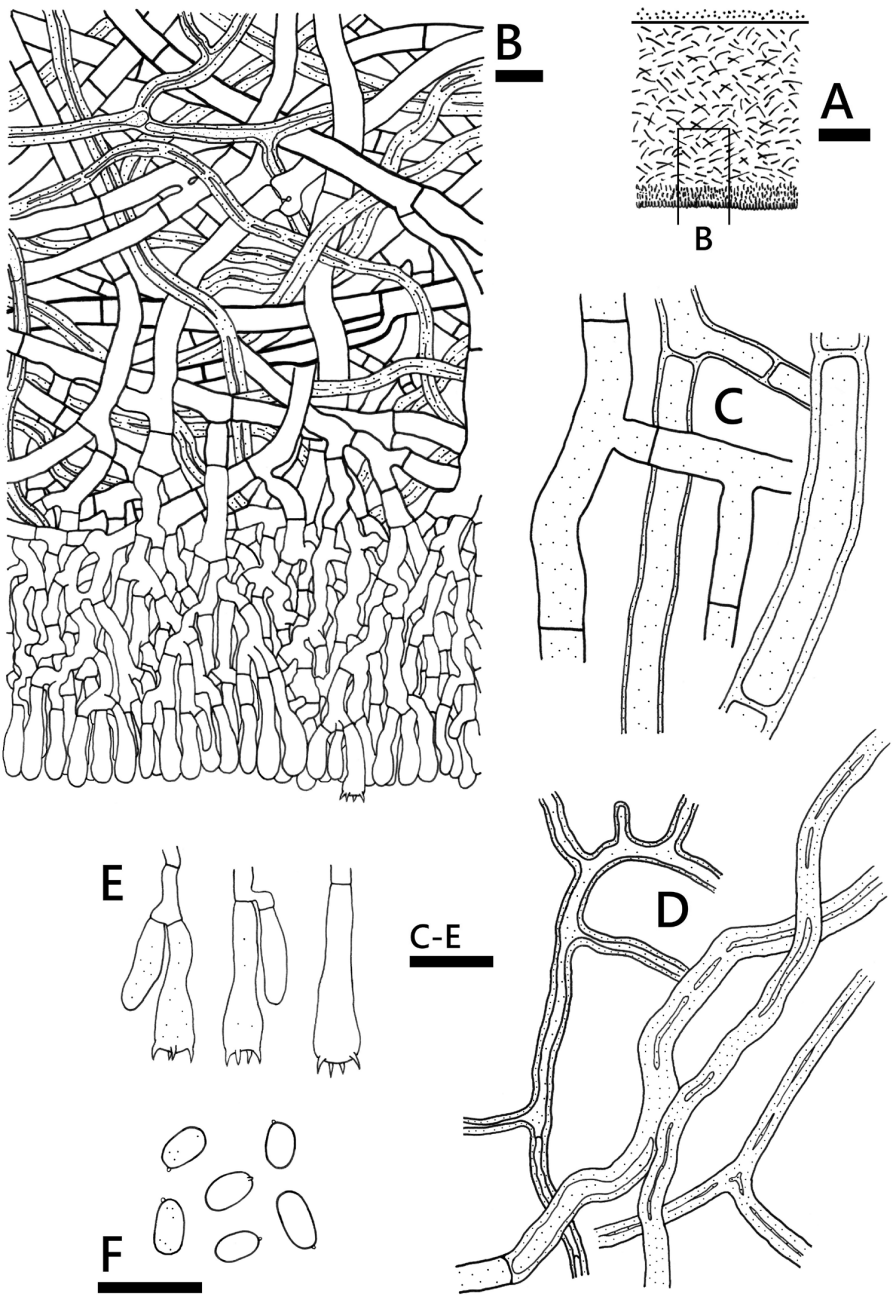
*Phanerochaetecanobrunnea* (holotype, *CHWC 1506*-*17*) **A** profile of basidiome section **B** lower part of basidiome section **C** generative hyphae **D** skeletal hyphae **E** basidia **F** basidiospores. Scale bars: 100 μm (**A**); 10 μm (**B–F**).

#### Phanerochaete cystidiata

Taxon classificationFungiPolyporalesPhanerochaetaceae

Sheng H. Wu, C.C. Chen & C.L. Wei sp. nov.

MycoBank No: 827412

Figs 3B , 5

##### Diagnosis.

*Phanerochaetecystidiata* is characterised by having a fibrillose margin of the basidiome and apically narrow or tapering leptocystidia that are more or less encrusted. Additionally, crystal masses are present in the hymenial layer.

##### Holotype.

TAIWAN. Nantou County: Aowanta, 23°57'N, 121°10'E, 1200 m alt., on angiosperm branch, coll. C.C. Chen, 28 Aug 2017, *GC 1708-358* (TNM F0031801).

##### Etymology.

From cystidiatus, referring to the presence of cystidia of this species.

##### Description.

Basidiome resupinate, effuse, adnate, membranaceous, 120–250 (–330) μm thick in section. Hymenial surface creamish-yellow, brownish in KOH, smooth to occasionally slightly tuberculate (due to crystal masses in hymenial layer), sometimes cracked; margin whitish or concolorous, fibrillous to fimbriate, occasionally determinate.

Hyphal system monomitic; hyphae simple-septate, clamp connections rarely present in subiculum. Subiculum fairly uniform, with somewhat loose to fairly dense texture, usually very dense near the substrate, 70–150 μm thick; hyphae more or less horizontal, colourless, fairly straight, moderately ramified, occasionally strongly encrusted with crystals, 3–6 (–7) μm diam., with 0.8–1.5 μm thick walls, anastomoses occasional. Hymenial layer thickening, with fairly dense texture, 50–100 (–180) μm thick, occasionally stratified; hyphae more or less vertical, colourless, 2.5–5 μm diam., thin-walled. Crystal masses occasionally abundant in hymenial layer. Leptocystidia numerous, immersed or emergent, cylindrical, median part usually slightly swollen and slightly thick-walled, with narrow or tapering apices, sparsely to heavily encrusted, (35–) 40–60 × 4–5.5 μm. Basidia subclavate to narrowly clavate, usually guttulate when mature, 20–30 × 4.5–5.5 μm, 4-sterigmate. Basidiospores ellipsoid to narrowly ellipsoid, adaxially flattened, smooth, thin-walled, guttulate, IKI–, CB–, mostly 4–5.3 × 2.5–3 μm. [4–5 (–5.5) × (2.5–) 2.7–3 (–3.3) μm, L = 4.59±0.43 μm, W = 2.86±0.18 μm, Q = 1.61 (n = 30) (*GC 1708-358*); (4–) 4.2–5 (–5.5) × 2.5–3 (–3.2) μm, L = 4.72±0.40 μm, W = 2.79±0.20 μm, Q = 1.70 (n = 30) (*Wu 1708-326*)].

##### Additional specimens examined (paratypes).

CHINA. Yunnan Province: Wenshan Zhuang and Miao Autonomous Prefecture, Maguan County, Dalishu Township, Lake, 23°07'04"N, 104°08'17"E, 1800 m alt., on angiosperm branch, coll. C.C. Chen, 7 Aug 2017, *GC 1708-76* (TNM F0031803). TAIWAN. Nantou County: Aowanta, 23°57'N, 121°10'E, 1200 m alt., on angiosperm branch, coll. S.H. Wu, 28 Aug 2017, *Wu 1708-326* (TNM F0031802).

##### Distribution.

Known from China (Yunnan Province) and Taiwan (type locality).

##### Remarks.

*Phanerochaeteericina* is the most closely related species (Figs 1, 2), but differs in having brownish hymenophore, frequently branched narrow hyphae (quasi-binding hyphae) and cystidia that are not encrusted (Wu 1990). *Phanerochaeteburtii* (Romell) Parmasto, *P.carnosa* (Burt) Parmasto, *P.calotricha* (P. Karst.) J. Erikss. & Ryvarden, *P.citrinosanguinea* Floudas & Hibbett, *P.pseudosanguinea* Floudas & Hibbett, *P.sanguinea* (Fr.) Pouzar and *P.sanguineocarnosa* Floudas & Hibbett also have a more or less fimbriate margin of the basidiomes, apically narrow or tapering cystidia and similar-sized basidiospores; however, their cystidia are not or only rarely encrusted. These species form a strongly supported monophyletic group, while *P.cystidiata* is phylogenetically distantly related to this group (Figs 1, 2).

**Figure 5.**
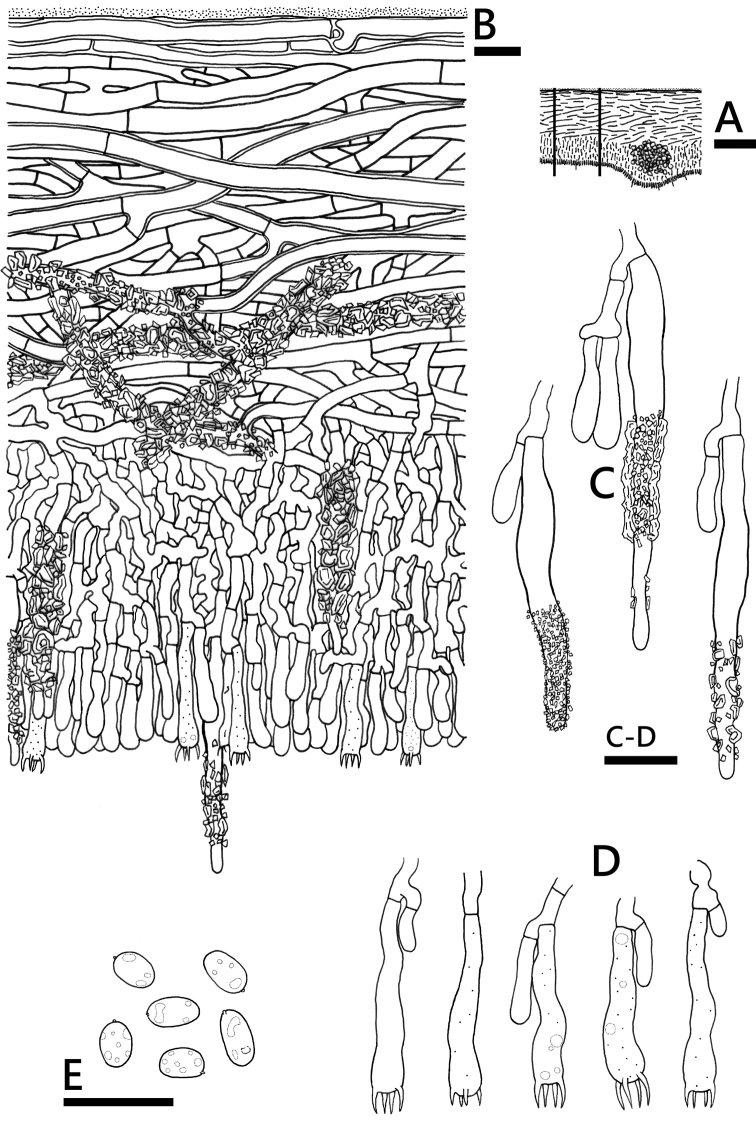
*Phanerochaetecystidiata* (holotype, *GC 1708-358*) **A** profile of basidiome section **B** basidiome section **C** leptocystidia **D** basidia **E** basidiospores. Scale bars: 100 μm (**A**); 10 μm(**B–E**).

#### Phanerochaete fusca

Taxon classificationFungiPolyporalesPhanerochaetaceae

Sheng H. Wu, C.C. Chen & C.L. Wei sp. nov.

MycoBank No: 827413

Figs 3C , 6

##### Diagnosis.

*Phanerochaetefusca* is characterised by smooth to tuberculate dark brown hymenial surface, monomitic hyphal system with brown subicular hyphae and leptocystidia with narrow or tapering apices. Additional diagnostic features: hyphae and cystidia usually with adventitious septa, subicular hyphae sometimes swollen at hyphal ends and basidia becoming thick-walled and brownish when old.

##### Holotype.

CHINA, Hubei Province: Shennongjia Forest Area, Wenshui Forest Farm, 31°44'N, 110°20'E, 1700 m alt., on angiosperm branch, coll. S.H. Wu, 19 Sep 2014, *Wu 1409*-*161* (TNM F0029722).

##### Etymology.

From fuscus (= dark brown), referring to the colour of the hymenial surface.

##### Description.

Basidiome resupinate, effuse, adnate, membranaceous, 250–580 μm thick in section. Hymenial surface dark brown, slightly darkening in KOH, smooth to tuberculate, not cracked; margin concolorous, more or less separable, determinate.

Hyphal system monomitic; hyphae simple-septate, clamp connections rarely present in subiculum. Subiculum fairly uniform, with dense texture, 200–480 μm thick; hyphae more or less horizontal, brown, fairly straight, moderately ramified, usually swollen at hyphal ends, usually encrusted near subhymenium, (2.5–) 3–7 (–7.5) μm diam., with slightly thick to up to 2 μm thick walls, with small oily drops, usually with adventitious septa. Hymenial layer thickening, with dense texture, 50–100 μm thick; hyphae more or less vertical, brownish to subcolourless, 2.5–4 μm diam., slightly thick-walled. Leptocystidia numerous, originating from hymenial layer, projecting, cylindrical with narrow or tapering apices, sometimes encrusted, subcolourless to brownish, usually with 1 or 2 adventitious septa, 50–70 × 3.5–5.5 (–6) μm, with thin to up to 1 μm thick walls. Basidia clavate or occasionally narrowly clavate, subcolourless to brownish, sometimes with an adventitious septum, 22–50 × 5–6 μm, with thin to up to 1 μm thick walls, 4-sterigmate. Basidiospores narrowly ellipsoid to subcylindrical, adaxially slightly concave, smooth, thin- to slightly thick-walled, colourless to sometimes brownish, IKI –, CB –, mostly 5.7–7.3 × 3–3.5 μm. [(5.3–) 5.7–7.3 (–7.8) × (2.8–) 3–3.5 (–3.7) μm, L = 6.63±0.64 μm, W = 3.24±0.28 μm, Q = 2.05 (n = 30) (*Wu 1409-161*)].

##### Additional specimen examined (paratype).

CHINA. Hubei Province: Shennongjia Forest Area, Wenshui Forest Farm, 31°44'N, 110°20'E, 1700 m alt., on angiosperm branch, coll. S.H. Wu, 19 Sep 2014, *Wu 1409*-*163* (TNM F0029723).

##### Distribution.

Known from China (Hubei Province).

##### Remarks.

*Phanerochaetestereoides* Sheng H. Wu resembles *P.fusca* in having brown subicular hyphae and leptocystidia. However, hymenial surface of the former is pale greyish-brown, while the latter is dark brown. Moreover, cystidia of *P.stereoides* are uniformly thin-walled and colourless, not with 1 or 2 adventitious septa. These two species are not closely related according to the phylogenetic analyses (Fig. 2). *Phanerochaeteporostereoides* is the most closely related species (Fig. 2). Like *P.fusca*, it has brown subicular hyphae, but differs by lacking cystidia and by smaller basidiospores [(4.5–) 4.7–5.3 (–5.5) × (2.3–) 2.5–3.1 (–3.3) μm], according to Liu and He (2016).

**Figure 6.**
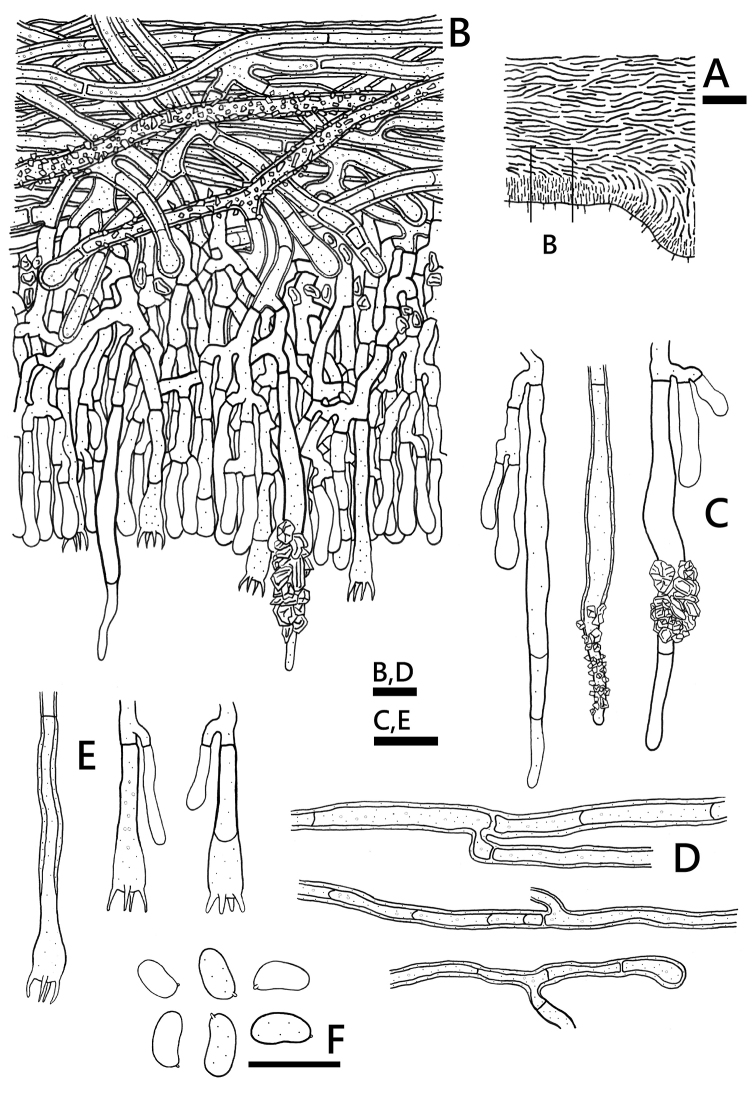
*Phanerochaetefusca* (holotype, *Wu 1409*-*161*) **A** profile of basiome section **B** basidiome section **C** leptocystidia **D** subicular hyphae, usually swollen at hyphal ends **E** basidia **F** basidiospores. Scale bars: 100 μm (A); 10 μm (**B–F**).

## Supplementary Material

XML Treatment for Phanerochaete canobrunnea

XML Treatment for Phanerochaete cystidiata

XML Treatment for Phanerochaete fusca
